# Generation of open biomedical datasets through ontology-driven transformation and integration processes

**DOI:** 10.1186/s13326-016-0075-z

**Published:** 2016-06-03

**Authors:** María del Carmen Legaz-García, José Antonio Miñarro-Giménez, Marcos Menárguez-Tortosa, Jesualdo Tomás Fernández-Breis

**Affiliations:** Departamento de Informática y Sistemas, Universidad de Murcia, IMIB-Arrixaca, Murcia, 30071 Spain; Institute of Medical Informatics, Statistics and Documentation, Medical University of Graz, Graz, 8036 Austria

**Keywords:** Semantic web, Ontologies, Biomedical open data, Data transformation

## Abstract

**Background:**

Biomedical research usually requires combining large volumes of data from multiple heterogeneous sources, which makes difficult the integrated exploitation of such data. The Semantic Web paradigm offers a natural technological space for data integration and exploitation by generating content readable by machines. Linked Open Data is a Semantic Web initiative that promotes the publication and sharing of data in machine readable semantic formats.

**Methods:**

We present an approach for the transformation and integration of heterogeneous biomedical data with the objective of generating open biomedical datasets in Semantic Web formats. The transformation of the data is based on the mappings between the entities of the data schema and the ontological infrastructure that provides the meaning to the content. Our approach permits different types of mappings and includes the possibility of defining complex transformation patterns. Once the mappings are defined, they can be automatically applied to datasets to generate logically consistent content and the mappings can be reused in further transformation processes.

**Results:**

The results of our research are (1) a common transformation and integration process for heterogeneous biomedical data; (2) the application of Linked Open Data principles to generate interoperable, open, biomedical datasets; (3) a software tool, called SWIT, that implements the approach. In this paper we also describe how we have applied SWIT in different biomedical scenarios and some lessons learned.

**Conclusions:**

We have presented an approach that is able to generate open biomedical repositories in Semantic Web formats. SWIT is able to apply the Linked Open Data principles in the generation of the datasets, so allowing for linking their content to external repositories and creating linked open datasets. SWIT datasets may contain data from multiple sources and schemas, thus becoming integrated datasets.

## Introduction

Biomedicine is a knowledge based discipline, in which the production of knowledge from data is a daily activity. Current biomedical research generates an increasing amount of data, whose efficient use requires computing support. Traditionally, biomedical data have been stored in heterogeneous formats in various scientific disciplines. Since the development of the Protein DataBank [1] in the seventies, life scientists have developed many biological databases, and there are more than 1500 biological databases according to the 2015 Molecular Biology Database Update [2]. As a consequence, biological data are represented in disparate resources [3], which makes data retrieval and management hard for life scientists because they are required to know: (1) which resources are available and contain the desired information; (2) the meaning of the data types and fields used in each resource; and (3) how such resources can be accessed and queried. There is, therefore, a clear need for facilitating the integrated use of such resources. Unfortunately, there is also heterogeneity in the formats used for storing such data, since they are not usually the most machine-friendly ones [4].

On the medical and clinical side, the advent of electronic health records (EHRs) contributes to making more data available for computer processing, but suffers from similar problems. The heterogeneity of EHR systems can be assimilated to the one of biological databases. The semantic interoperability of EHR data not only has been identified as a need but also considered as a reason for inefficiencies within the healthcare system [5, 6] and for the waste of billions of dollars in the United States annually [7].

Translational research aims at applying basic biological results and data into clinical activities and routine. In recent years, supporting data-driven medicine has been set as a challenge for translational bioinformatics [8]. For this purpose, the integration and joint analysis and exploitation of heterogeneous data, both biological and medical becomes critical, hence, solutions in this area are required.

In the technical side, the Semantic Web [9] describes a new form of Web content meaningful to computers, in which the meaning is provided by ontologies. An ontology represents a common, shareable and reusable view of a particular application domain [10]. The fact that machines know the meaning of content enables the use of automated reasoning, which permits to infer new information or to check the logical consistency of the content. The Semantic Web has been proposed as a technological space in which biomedical data can be integrated and exploited [11]. The growing interest of the biomedical community in the Semantic Web can be illustrated by the fact that repositories such as BioPortal [12] contain at the time of writing more than 500 biomedical ontologies, controlled vocabularies and terminologies.

The Semantic Web community wishes to achieve the Web of Data, which would semantically connect datasets distributed over the Internet. The Linked Open Data (LOD) effort^1^ pursues the publication and sharing of biomedical datasets using semantic formats such as RDF^2^ or OWL^3^. The biomedical community is heavily involved in the development of the LOD cloud [13], since integration and interoperability are fundamental for biomedical data analysis [14]. The LOD cloud offers a promising infrastructure for such a goal. The availability of consensus ontologies generated by the biomedical community facilitates the publication of data in the LOD cloud, since those ontologies can be used as vocabularies for the RDF datasets. Most efforts in this area have been solved by in-house solutions, implementing resource-specific transformation scripts. Hence, we believe that there is a need for methods and tools that contribute to standardise the process of getting biomedical datasets in semantic formats.

Since the development and success of the Gene Ontology [15], ontologies have been used to support data annotation processes. The development and evolution of the Semantic Web technologies has permitted to increase the variety of use of such technologies in biomedical domains. In the area of biomedical databases we can point out two efforts of particular significance. First, the European Bioinformatics Institute (EBI) has developed an RDF platform which permits the semantic exploitation of the content of a series of EBI resources, including UniProt [16]. Second, the Bio2RDF initiative [13] has created RDF versions of thirty five biomedical resources (Release 3 July 2014). These efforts pursue the development of the biomedical LOD. In the area of EHRs, the SemanticHealth project identified that ontologies should play a fundamental role for the achievement of the semantic interoperability of EHRs [6]. Since then, Semantic Web technologies have been increasingly applied in the EHR domain with different purposes: representation of clinical models and data [17–19]; interoperability of models and data [20–22]; application of quality measurements and protocols to data [23, 24].

The main objective of the present work is to propose a method that could serve to simplify the process of generating integrated semantic repositories from heterogeneous sources. The approach will be able to work with relational databases, XML documents, and EHR data and will produce datasets described by means of ontologies. The transformation process is independent of the formalism used for capturing the data to be transformed. This process will be driven by the semantics of the domain to ensure the correctness and logical consistency of the resulting content. This will be achieved by defining mappings between the data schemas and the ontologies, which will provide the semantic content. Our approach will be able to create a repository from multiple sources, which will require to define mechanisms for merging the data about the same entity contained in the different resources. Besides, the resulting content will be generated according to the principles of Linked Open Data. We will also describe our Semantic Web Integration Tool (SWIT), which implements the transformation and integration methods, and the application of our method in different use cases. The expected contributions of our research are (1) the common transformation and integration process for heterogeneous biomedical data; (2) enabling the design of reusable mappings between schemas driven by domain knowledge; (3) the application of Linked Open Data principles to generate interoperable, semantically-rich, open, biomedical datasets.

## Background

### Biomedical data

The term biomedical data covers a wide range of types of data used in biomedicine. Such data are usually stored and represented in different, heterogeneous formats, which makes their joint exploitation difficult. In this work we are specially interested in the information contained in biomedical databases and in the content of electronic healthcare records because of their importance for biomedical and clinical research.

On the one hand, biomedical databases contain large volumes of complex, dynamic information about biomedical entities. The information about a concrete biomedical entity, like a protein, is distributed along many different databases, which makes necessary to combine information from different sources to get all the information. These heterogeneous resources do not even share identifiers for the biological entities, although this particular aspect is being addressed by initiatives like identifiers.org [25]. XML files and relational databases are popular formats used for the representation and sharing of biomedical databases. For instance, OrthoXML and SeqXML [26] are two XML formats to standardise the representation of orthology data. Relational databases have gained popularity in the last years because they are effective in retrieving data through complex queries. Biomedical resources such as the Gene Ontology [15] or CHEBI [27] provide their data in relational format.

On the other hand, the electronic health record of a patient stores all the information digitally recorded from the interactions of the patient with the health system. In the last decades, many efforts have addressed the development of EHR standards and specifications, such as HL7 [28], openEHR [29], and ISO EN 13606 [30]. Such standards and specifications are based on the dual model architecture, which distinguishes two modelling levels. On the one hand, the information model provides the generic building blocks to structure the EHR information. On the other hand, clinical models are used to specify clinical recording scenarios by constraining the information model structures. In both openEHR and ISO EN 13606, clinical models are named archetypes and they have been considered a promising way of sharing clinical data in a formal and scalable way [5]. Archetypes are used to specify clinical recording scenarios. An archetype may be used to record clinical data about a laboratory test, a blood pressure measurement, a medication order, etc. They constitute a standardised way of capturing clinical data according to the archetype model [31]. They are usually defined in the Archetype Definition Language (ADL)^4^. EHR data extracts are usually represented as XML documents, whose content should satisfy the constraints specified in the archetype.

The joint semantic exploitation of data stored in XML files or in relational databases requires methods for the transformation of the data into semantic formats. Both XML technologies and relational databases provide schemas which define the structure of the datasets. In our approach, such schemas will be used to define generic processing methods able to transform and exploit XML and relational data using semantic technologies. More concretely, XML schemas, ADL archetypes and the schema of relational databases will be managed in our approach. In practical terms, ADL archetypes play the role of XML Schemas.

### Semantic representation and access to biomedical data

The World Wide Web Consortium has developed a series of Semantic Web standards for exchanging data (e.g., RDF), for representing their semantics (e.g., OWL) and for querying these data (e.g., SPARQL^5^). Automated reasoners (e.g., Hermit [32], Pellet [33]) can be used in conjunction with Semantic Web content to check for the consistency of data or to infer new information. Semantic Web technologies also offer mechanisms for storing semantic data called triplestores and whose performance for complex queries is continuously improving [34]. Linked Open Data is a Semantic Web initiative aiming to materialise the Web of Data through the publication and sharing of datasets using semantic formats. Linked Open Data datasets meet four requirements [35]: (1) use URIs as names for things; (2) use HTTP URIs so that people can look up those names; (3) when someone looks up an URI, provide useful information, using the Semantic Web Standards like RDF and SPARQL; and (4) include links to other URIs, so related things can be discovered.

The data published in the Linked Open Data (LOD) cloud are diverse in granularity, scope, scale and origin, and the LOD cloud is constantly growing with information from new domains. Berners-Lee^6^ suggested a five-star deployment scheme for Open Data, where each level imposes additional conditions. The use of RDF and an appropriate use of URIs permit the achievement of four-stars datasets. The fifth one can be achieved by linking your dataset to external ones. It should be noted that the community is trying to impose additional conditions to get such stars [36]. The number of biomedical datasets in the LOD cloud is still reduced in comparison with the number of existing biomedical databases, but our approach aims at facilitating biomedical communities to join and follow the LOD principles and effort. We believe that the development of methods that permit to get five-star datasets would contribute to the development of the Semantic Web.

Next, we describe the two major approaches for data exploitation using semantic technologies: (1) the transformation of data into semantic formats; and (2) ontology-based data access, which works on traditional formats.

#### Semantic transformation of biomedical data

Data transformation methods have been traditionally used in projects that use the data warehouse approach and OLAP for the semantic exploitation of data [37], and with both XML datasets and relational databases. On the XML side, [38] presented an approach that transforms XML elements into RDF statements, but does not transform XML attributes. Another approach^7^ transforms XML instances into RDF according to a mapping between XSD and OWL^8^. These XSD2OWL mappings are canonical, since all the XML files are transformed into RDF by applying the same rules. Canonical XSLT-based approaches have also been proposed [39, 40]. More recently, [41] proposed the transformation of XML into RDF by applying XPath-based mappings. On the relational database side, the W3C RDB2RDF specification^9^ proposes a canonical transformation/mapping for relational databases to RDF. Such a transformation can be considered a change of format, because the real meaning of the entities represented is not used in such a process. This is an important limitation we find in the state of the art transformation approaches and tools, since they do not take into account the underlying model of meaning.

In the last years, Bio2RDF has become the most prominent initiative for the generation of biomedical RDF datasets. Bio2RDF has developed RDF versions for 35 datasets (Release 3 July 2014), and its website contains non-canonical transformation scripts for such resources. To the best of our knowledge, the links between the data and the domain knowledge are not made explicit in such transformation scripts. For instance, there is no guarantee that content about a protein from different resources is transformed using the same meaning, and this makes more difficult to expand the approach and to find errors.

From a process perspective, the semantic transformation of data requires the execution of Extraction-Transformation-Load (ETL) processes. Canonical transformation approaches apply the same ETL process to all the data. The required information about the semantics of the data sources is sometimes missing in the data schema or coded in natural language [42], which makes such canonical processes not effective enough to obtain semantically-rich datasets. Ontology-driven ETL processes use ontologies for giving precise meaning to the source data, which will be made explicit in the transformation phase. This also enables consistency checking in the transformation and/or load phases, which prevents from the creation of logically inconsistent content. Tools like RDB2OWL [43] and Karma [44] are examples of tools that exploit mappings between relational schemas and ontologies to generate RDF content.

#### Ontology-based data access

Ontology-Based Data Access (OBDA) permits to exploit repositories in traditional formats using semantic technologies. As stated in [45], the underlying idea is to facilitate access to data by separating the user from the raw data sources. In OBDA, an ontology provides the user-oriented view of the data and makes it accessible via queries formulated solely in semantic languages such as SPARQL. In OBDA approaches, a mapping between the ontology and the data sources defines the view on the source data that can be exploited using semantic technologies.

Different OBDA approaches for accessing XML and relational data can be found in the literature. On the XML side, XSPARQL^10^ was proposed as a query language combining XQuery and SPARQL for data transformation between XML and RDF, and XS2OWL [46] creates OWL ontologies from XML schemas for allowing querying XML data using SPARQL queries. On the relational databases side, Triplify [47], D2RQ [48], Virtuoso [49], Quest [50], Ultrawrap [51] and Ontop [52] are likely to be the most popular OBDA systems nowadays. Such systems differ in how they express the mappings, how they translate the queries and in the reasoning capabilities. Current OBDA approaches are limited in their support for reasoning. For example, D2RQ does not support reasoning and OWL2 QL is the level of reasoning offered by Ontop. OBDA tools are starting to provide support to rule languages such as SWRL^11^ for enabling users to exploit Semantic Web rules over data in traditional formats. Given that our approach will rely on reasoning for guaranteeing the consistency of the transformation and integration of data, OBDA is not the best option for our work.

### Integration of biomedical data

A variety of XML-based approaches for the integration of data are presented in [53]. The main conclusion of such study is that XML has succeeded in the integration of data, and has opened new opportunities for research, but the variety of XML-based data formats makes very difficult the effective integration of data sources. The solution proposed is the adoption of semantic formats, which leads us to semantic data integration scenarios, in which ontologies ideally provide the global schema. When this happens, the integration process can also take advantage of the benefits described for ETL processes such as the use of precise meaning or consistency checking. This semantic approach is also supported by the fact that the Semantic Web is a natural space for the integration and exploitation of data [11].

There are four major types of data integration architectures [53]: data warehouse, mediator-based, service-oriented and peer-based. The data warehouse approach is more related to the semantic transformation methods, and the other three are more related to ODBA, since they perform a virtual integration. In the literature, we can find biomedical semantic integration approaches such as Ontofusion [54] or TAMBIS [55], which fall in the area of mediator-based systems or OGO [56], which follows the data warehouse approach. Bio2RDF uses a data integration approach based on links. This is a case of virtual integration that uses *owl:sameAs* statements to identify instances referring to the same entity in other resources.

One limitation of state of the art approaches and tools is that they are not generic enough in the sense of their applicability to both XML and relational data. Data integration has to overcome issues such as redundancy or inconsistency between the data sources. Most mediator or link-based approaches aggregate the data from the different sources, but the availability of mechanisms for preventing redundancy or inconsistency is not common. Those mechanisms are easier to include in data warehouse-oriented methods, which provide more control on the data. Our approach will be mostly based on data warehouse, since the integrated datasets (from XML and relational resources) are assumed to be created in a common repository. Besides, in order to preserve the original datasets in the integrated, semantic repository, the configuration of the integration process will enable to merge those equivalent instances or linking them through *owl:sameAs* statements.

## Methods

**Fig. 1:**
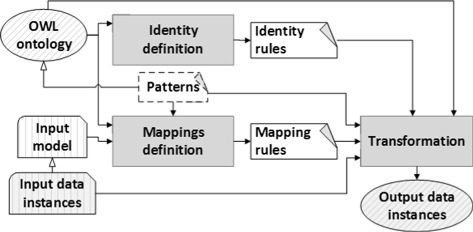
Overview of the transformation approach

In this section we describe the methods included in our approach for the generation of the open biomedical datasets. Figure 1 provides a generic description of the method for a single input data resource. Our data transformation approach is based on the definition of rules between an input schema and an OWL ontology. Once defined the mapping rules, the transformation approach may also take into account identity rules defined over the OWL ontology. Identity rules establish which properties permit identifying an individual of a certain ontology class. Thus, these rules permit to merge different individuals of the same class. Besides, the transformation method will be able to detect and, therefore, avoid the creation of logically inconsistent content by checking the consistency of the OWL ontology. This is done because the whole process is supported by OWL ontologies and, therefore, automated reasoning techniques can be applied. In general, the approach can be applied to any input data model providing entities, attributes and relations. In this work, we will use XML and relational databases as input data models. A practical requirement for our approach is that the input schema and the ontology should have some domain content in common. In addition to this, the output data instances shown in Fig. 1 can be expressed in RDF or OWL.

### Data transformation rules

The transformation rules define how the content of the input dataset is transformed into a semantic format, and play two major roles: (1) controlling that the information represented according to the input schema is correctly transformed into the semantic format; and (2) preventing redundancy in the set of output dataset. For this purpose, two major types of rules are defined in our approach, namely, mapping rules and identity rules. Both are described in the next subsections.

#### Mapping rules

**Fig. 2:**
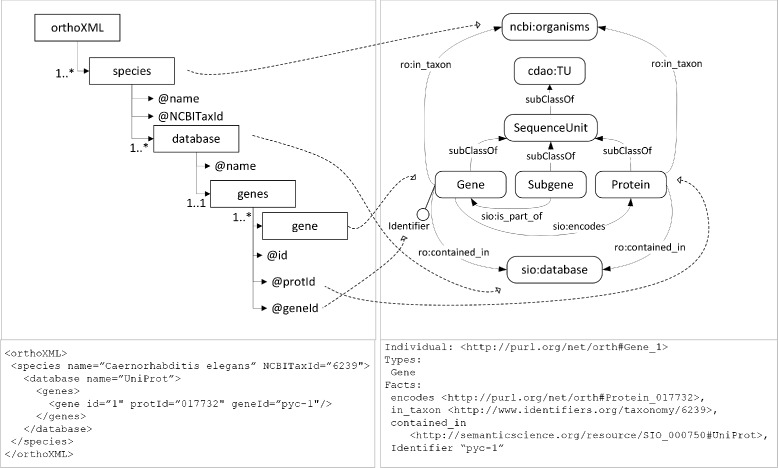
Description of the mapping between the OrthoXML schema (*left*) and the orthology ontology (ORTH)(*right*), where dashed lines represent the links between the content of the OrthoXML schema and the ontology and corresponding instances that fulfill the relation of congruence

The definition of the mapping rules will be illustrated with the example described in Fig. 2. In this example, (1) the input schema is OrthoXML [26] (Figure 2 top left), which is a standardised format for the representation of information about orthologous genes, (2) the ontology is the Orthology Ontology (ORTH)^12^(Fig. 2 top right), which models domain knowledge about orthology. In the example, the entities of the input schema are represented with boxes, the attributes with @, and the relations with arrows. In the ontology, the classes are represented with rounded corner boxes, the datatype properties with a pin attached to the classes, and the object properties with arrows. Mapping rules link entities, attributes and relations of the input model with the ontology classes, datatype properties and object properties. In Fig. 2, dashed lines represent the mappings from the XML Schema to the ontology. For simplicity, this figure does not include mappings involving relations or object properties. The ontology contains a series of prefixes, which refer to ontologies reused in the ORTH: ro (Relations Ontology^13^), ncbi (NCBI Taxonomy^14^), cdao (Comparative Data Analysis Ontology^15^), and sio (Semanticscience Integrated Ontology^16^)

Generally speaking, the application of a mapping rule to an instance of the input dataset generates one individual in the ontology. The instance and the individual must fulfill a relation of congruence. For us, an ontology individual *t* is congruent with an input instance *s* if *t* can be obtained from *s* by applying a mapping rule and *t* is consistent with the axioms expressed in the ontology and with *s*. The consistency with the axioms expressed in the ontology has to be understood in OWL DL terms. The individual must be, in logical terms, a member of the class to which the membership is stated.

The bottom of Fig. 2 shows an input instance (left) and the result of transforming it into an ontology individual (right) by applying the corresponding mapping rules. The input instance is a gene with attributes protId=“O17732”, geneId=“pyc-1”, it is included in UniProt and it is associated with the species *Caenorhabditis elegans* (NCBITaxId=“6239”). The ontology individual *Gene_1* is a member of the class *Gene*, it has a datatype property *Identifier* with value “pyc-1”. It is linked to other individuals through the properties *encodes* (Protein_O17732), *ro:in_taxon* (6239), and *contained_in* (UniProt). These individuals are members of the classes *Protein*, *ncbi:organisms*, and *sio:database*, respectively. The ontology individual *Gene_1* is consistent with the ORTH ontology and consistent with the data defined for the input instance. Therefore, both entities are congruent.

Our approach requires transforming entities, attributes and relations, so the mapping rules must permit to achieve congruence at those three levels. To this end, three types of basic mapping rules have been defined:

**Entity rule**. It links an entity of the input schema with an OWL ontology class. It permits to create individuals in the OWL ontology. Let S be an entity of the input schema and T be a class of the target ontology. Then, the entity_rule(S, T) means that for every instance s of S, there is a congruent individual t which is an instance of T. For example, an entity rule in Fig. 2 serves to link the element *Gene* of the XML Schema and the class *Gene* in the ontology. An entity rule can be complemented by a conditional statement that transforms only those instances of S holding a certain condition on the value of a particular attribute. Using the classes of the previous example, let A1 be an attribute associated with S, and let c1 be a boolean condition over A1. The entity_rule(S, T, c1) means that for every instance s of the entity S fulfilling c1 there is a congruent individual t which is an instance of T. **Attribute rule**. It links an attribute of an entity of the input schema with the datatype property of an OWL ontology class. It permits to assign values to datatype properties in the ontology. Let S be an entity of the input schema, T an ontology class, and *A*_1_ and *A*_2_ attributes/datatype properties associated with S and T respectively. Then, the attribute_rule((S, *A*_1_), (T, *A*_2_)) means that for each instance of S associated with *A*_1_ from the same schema, there is a congruent individual of T associated with the datatype property *A*_2_ from the ontology and that *A*_1_ and *A*_2_ have the same value. For example, an attribute rule in Fig. 2 links the attribute *id* of the element *gene* in OrthoXML and the datatype property *Identifier* of the ontology class *Gene*. **Relation rule**. It links a relation associated with two entities of the input schema with an object property associated with two classes of the OWL ontology. Let *S*_1_ and *S*_2_ be entities of the input schema associated through *R*_1_ and *T*_1_ and *T*_2_ be classes of the ontology associated through the object property *R*_2_. Then, the relation_rule((*S*_1_, *R*_1_, *S*_2_), (*T*_1_, *R*_2_, *T*_2_)) means that given *S*_1_ and *S*_2_ linked through the relation *R*_1_, and given the entity_rule(*S*_1_, *T*_1_) and the entity_rule(*S*_2_, *T*_2_), then for each instance of *S*_1_ and *S*_2_ there will be individuals of *T*_1_ and *T*_2_ respectively that will be linked through *R*_2_. For example, a relation rule in Fig. 2 would link the hierarchical relation between *species* and *gene* in the XML Schema and the object property *ro:in_taxon* in the ontology.

#### Ontology transformation patterns

The previous basic rules do not support all the types of transformations needed in order to get semantically-rich datasets, because sometimes we need (1) to define rules that involve multiple input entities and one or many ontology classes, or (2) to add additional information to enrich the input data. Consequently, more complex transformations are needed. For this purpose we have adopted the ontology pattern approach. Our ontology transformation patterns represent a partial or complete semantic description of a class of the ontology. Patterns are intermediary entities among the input schema and the ontology from the perspective of the definition of mappings. Our patterns are templates designed by using OWL ontology classes, datatype properties, object properties and constraints. Such patterns have variables which are bound to the corresponding entities, attributes or relations.

A pattern can be defined as the tuple < S, V >, where S stands for the set of classes, datatype properties, object properties and individuals used in the pattern that are a subset of those defined in the OWL ontology, and V is the set of variables associated with the instances of classes or the values of properties in S. A pattern is instantiated by linking the variables with entities of the input schema, and can be used for creating new content in the OWL ontology. A pattern allows creating several new individuals, giving value to datatype properties and linking individuals through object properties. Moreover, a pattern can be reused several times acting as a template. A pattern also allows for specifying fixed content that does not depend on the input dataset or that cannot be obtained from it, so contributing to the semantic enrichment of the content.

**Fig. 3:**
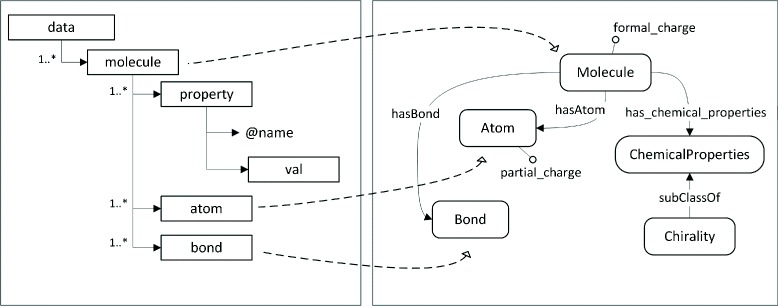
Description of the mapping between a XML schema and an ontology in the domain of molecules

Figure 3 shows an example of mapping between an XML Schema (left) that represents information about molecules and a molecule ontology (right). The ontology classes *Molecule*, *Atom* and *Bond* have a direct mapping with elements of the XML Schema but, for instance, the ontology does not have a class for representing chiral molecules. A chiral molecule can be defined as a molecule with the chemical property of chirality. In OWL, such definition can be represented as *Molecule and has_chemical_property some Chirality*. In the input schema, chirality is represented by the element *property* with attribute name *isChiral* and whose value is represented in the element *val*, whose value is 0 or 1. The pattern shown in Table 1, which is expressed in OPPL2^17^, defines the variable *?chiralMolecule* and such rule defines the axioms to be generated. The data instances with value 1 for the *isChiral* attribute (not shown in the table) are the input for this pattern.

**Table 1:** Definition of the pattern for ChiralMolecule

?chiralMolecule: INDIVIDUAL
BEGIN
ADD ?chiralMolecule instanceOf (Molecule and
has_chemical_properties some Chirality)
END;

We could have used basic mapping rules for defining the entity_rule(molecule, Molecule, on_condition(property (@name = “isChiral”)/val, 1))), which links molecules with value 1 for the *isChiral* property with the OWL Class Molecule. The OWL instances would be incomplete, because they would not contain information about chirality. That would make the input instances and the ontology individuals not congruent. The use of the pattern allows defining the mapping with the variable *?chiralMolecule*, and the additional, fixed information provided by the pattern permits to satisfy the congruence relation.

#### Identity rules

Identity rules define the set of datatype properties and object properties that permit to distinguish each individual in the ontology. These rules are useful to prevent the creation of redundant content and to support the integration of data from multiple sources, since identity rules permit to identify which entities with different URI, from the same or different datasets, represent the same entity.

Let *IR* be the set of datatype properties and object properties of the ontology that univocally defines the identity for the class C. The identity_rule(C, *IR*) means that all the individuals of C with the same value for the elements in *IR* are considered the same. We can define an identity rule for the class *Gene* using the datatype property *Identifier* and the object property 〈 Gene, ro:in_taxon, ncbi:organisms 〉. This identity rule means that two individuals of the class *Gene* (see Fig. 2), associated with the same individual of the class *ncbi:organisms* through the object property *ro:in_taxon*, and with the same value for the datatype property *Identifier* are the same individual.

#### The execution of the transformation process

The method runs the mapping rules in the following specific order:

- The basic entity rules are retrieved and executed. As a result of this step, a set of new individuals of each class of the ontology, written I, is generated.
- The group of patterns represents a special case, since they may generate new individuals (not obtained in the previous step) and may also add content to new generated ones. Therefore, the statement of the patterns that create new individuals are executed and those new individuals are added to I. The identification of which statements of the patterns generate new individuals is done by checking their definition.
- For each instance of the set I the process continues as follow:
  - The rest of statements of the patterns are executed to add any additional content to the individuals.
  - The basic attribute rules are retrieved and executed, so the values of the datatype properties of the individuals are assigned.
  - The basic relations rules are retrieved and executed, so the object properties are instantiated.
  - The identity rules are checked and, in case the instance is unique, it is added to the output dataset. Otherwise it is merged or linked to the equivalent one, depending on the behaviour defined for the rule.

### Data integration

#### The integration approach

Our approach for the integration of heterogeneous resources is based on the application of the transformation approach described above to the different resources, using the same OWL ontology as driver of the process. The construction of the integrated content requires mapping the schemas to the OWL ontology. The OWL ontology may have a series of ontology transformation patterns associated, which support the integration process. The use of patterns facilitates (1) reusing the transformation rules with different resources, and (2) overcoming the structural heterogeneity of input data schemas. Table 2 shows the pattern that defines a protein in the OWL ontology used in one of our use cases. This pattern not only avoids the user the need for knowing the structure of the ontology but also can be applied with minor modifications to data resources which store the relation protein-cds-transcript in different ways, or might even not be defined in the input schema. Table 3 shows how parametrizing the variable *?cds* from the variable *?protein*, the pattern can be applied to data resources with a direct protein-transcript relation without cds.

**Table 2:** Definition of the pattern for protein

?protein: INDIVIDUAL
?cds: INDIVIDUAL
?transcript: INDIVIDUAL
BEGIN
ADD ?protein instanceOf Polypeptide,
ADD ?protein derives_from ?cds,
ADD ?cds instanceOf CDS,
ADD ?cds part_of ?transcript,
ADD ?transcript instanceOf Transcript
END;

**Table 3:** Definition of the pattern for protein with minor modification for resources without CDS content

?protein: INDIVIDUAL
?cds: INDIVIDUAL = create(?protein.RENDERING+_CDS)
?transcript: INDIVIDUAL
BEGIN
ADD ?protein instanceOf Polypeptide,
ADD ?protein derives_from ?cds,
ADD ?cds instanceOf CDS,
ADD ?cds part_of ?transcript,
ADD ?transcript instanceOf Transcript
END;

#### The integration process

The integration of data is carried out through the transformation of each input resource. The mapping rules enable to generate OWL content, and the identity rules are applied during the transformation process to limit redundancy and to merge data instances. Their role is to identify which instances from different resources correspond to the same domain instance. Obviously, individuals with the same URI are considered the same one.

The integration method makes the following decisions concerning the usual problems in integration processes:

- Naming conflicts: Different input schemas may use different terms for the same element (i.e., entity, attribute, relation). The output OWL ontology provides the common vocabulary for the integrated repository, so the mappings from the different resources to the OWL ontology solve this problem.
- Data redundancy: More than one instance of the input resource may describe the same domain entity, so they are mapped to the same class in the OWL ontology. Identity rules permit to detect such situations and to merge or link the corresponding OWL data to minimise redundancy.
- Inconsistency due to incomplete data: The input schema may store less attributes and relations for a given entity than the OWL ontology. This may lead to an inconsistent OWL knowledge base in case the identity rules cannot be checked. When such situation is detected, the corresponding source data are not transformed, so inconsistencies are prevented. Patterns providing values for missing data to such identity properties could be used to overcome this situation.
- Differences between the resources: It may happen that an OWL individual is created by using different instances of the data resources, which may have different values for common attributes or relations. This may be an issue for properties associated with the identity rules. In such case, they are considered different individuals, which are created unless they would make the knowledge base inconsistent.

## Results

In this section we describe the main results of our work. First, we will describe the tool that implements the transformation approach. Second, we will describe how the tool has been used in different biomedical scenarios.

### The SWIT tool

The transformation approach has been implemented in a web tool called SWIT^18^. SWIT provides a web interface through which the user is guided to perform all the steps of the process. SWIT is currently supporting MySQL databases, XML schemas and ADL archetypes as input schemas. SWIT permits to generate the output dataset in OWL or RDF formats, which can be downloaded or directly stored in Virtuoso [49] or in a Jena knowledge base^19^.

The user can define the mappings between the input schema and the OWL ontology. For this purpose, mappings created in other transformation processes can be uploaded and reused. Once the mappings have been defined, they can be executed, thus generating the corresponding RDF/OWL content. SWIT applies the mapping rules to the data source to generate the semantic content, checking the identity rules to guarantee that redundant individuals are not created. This process also uses automated reasoning to ensure that only logically consistent content is transformed. SWIT uses both the OWLAPI [57] and the Jena API for processing and generating the RDF/OWL content, Hermit [32] as reasoner, and the patterns are implemented using OPPL2.

**Fig. 4:**
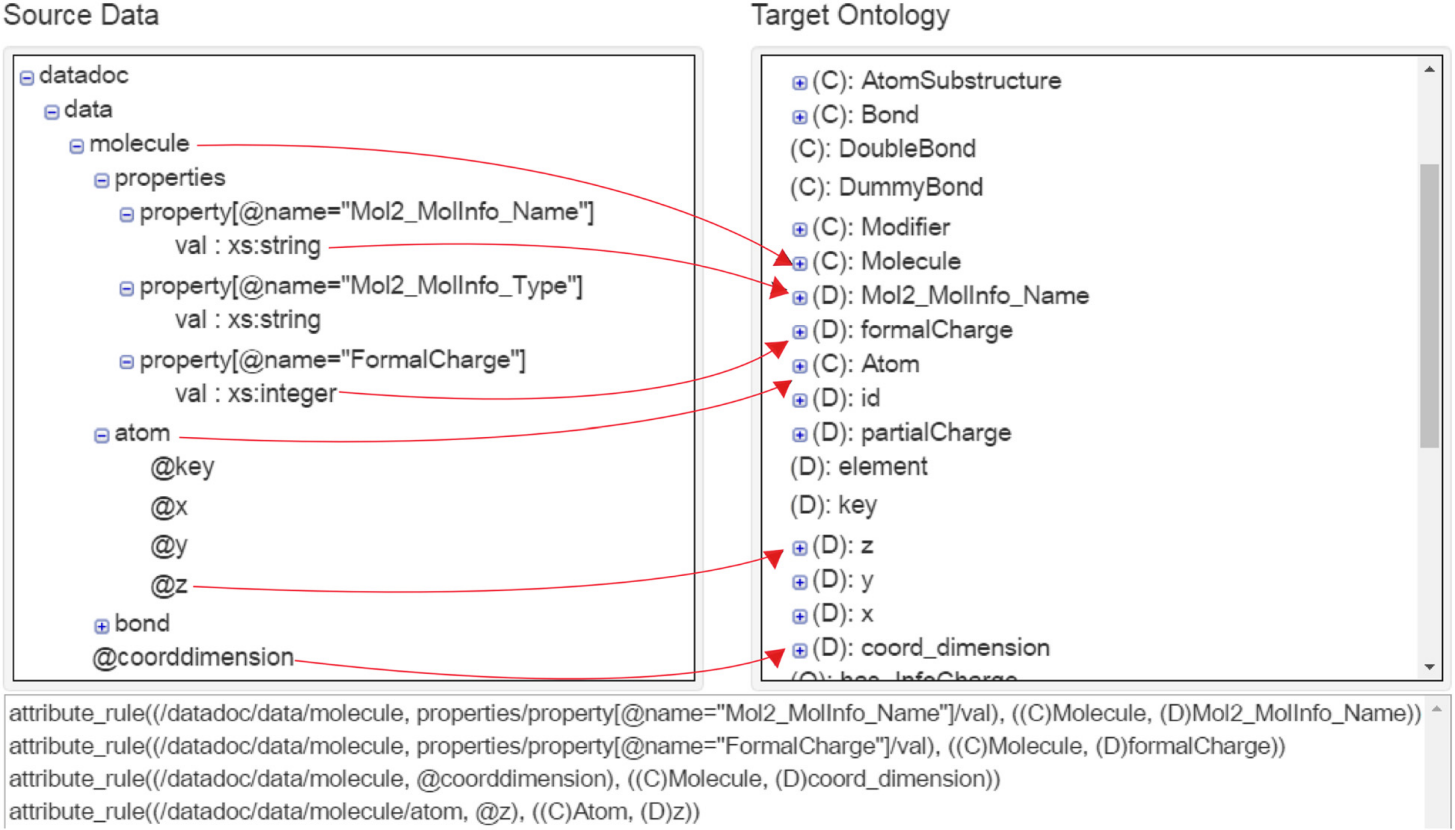
Mapping interface of SWIT: (*left*) part of an XML schema about molecules; (*right*) part of the classes and properties of domain ontology; (*bottom*) excerpt of the mappings defined between the XML schema and the ontology

Figure 4 shows a part of the mapping interface, which has three main parts. The left side shows the input schema using a hierarchical representation. The right side corresponds to the OWL ontology. The lower part of the figure is a text box, which contains the mapping rules defined. For example, the third line defines the mapping of the attribute *coorddimension* of the entity *molecule* to the datatype property *coord_dimension* of the ontology class *Molecule*.

**Fig. 5:**
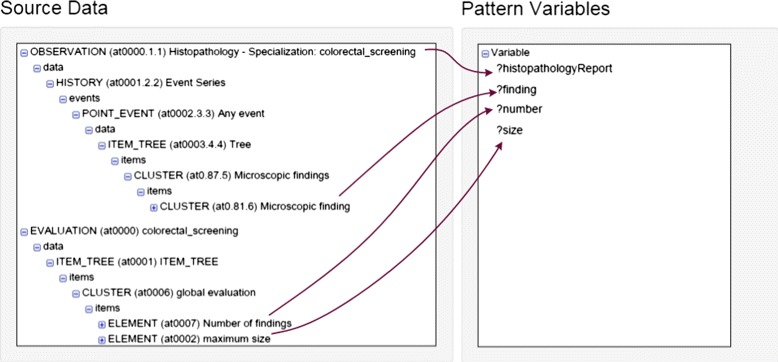
Example of pattern mapping in SWIT

Figure 5 is a screen snapshot of the definition of the mapping of entities of the input schema to a transformation pattern. In this case the input schema consists on openEHR archetypes (left), which are mapped onto an ontology transformation pattern for histopathology reports. In the figure, we can see that the mapping would associate a particular element of the archetypes with each variable of the pattern. In this case, the expression corresponding to the mapping rule is not shown in the figure.

### Data transformation use cases

In this section we explain how we have used SWIT in three biomedical domains. Our website contains more information about these efforts, including the mapping files and examples of content generated.

#### Orthology data

We have used SWIT to generate the integrated OGOLOD Linked Open Dataset [58]. The first version of OGOLOD was created with the purpose of providing an integrated resource of information about genetic human diseases and orthologous genes, given the increasing interest in orthologs in research [59]. OGOLOD integrates information from orthology databases such as Inparanoid [60] and OrthoMCL [61], with the OMIM database [62]. The content of OGOLOD was generated using a method to transform the content of relational databases into an RDF repository. The OGOLOD repository uses the OGO ontology [56] as a scaffold to link the integrated information about orthologs and diseases.

SWIT is currently being used to support the standardisation of orthology content^20^ [63] promoted by the Quest for Orthologs consortium^21^. For this purpose, OrthoXML [26] is the input schema. OrthoXML defines a standardised XML schema that provides the elements to describe an orthology relationship in a uniform way, and this format is being used by a number of orthology databases. We have defined and executed the corresponding mapping rules between OrthoXML format and the Orthology Ontology (ORTH) using SWIT. So far, this has permitted to generate an integrated dataset containing more than 2 billion triples.

#### EHR data

SWIT was the tool used for transforming EHR data of colorectal cancer patients in the study described in [24]. The study was performed with real anonymised data from the Colorectal Cancer Program of the Region of Murcia and included data from more than two thousand patients. This work required to transform data from a proprietary format to a semantic format in order to apply colorectal cancer protocols to the patient data. Such protocols were applied using automatic reasoning over the semantic content to determine the level of risk of each patient. SWIT was the tool employed in the processing and transformation of the EHR data once transformed from the proprietary format into openEHR XML extracts. In this case, those extracts and archetypes were the source data and input schema for SWIT. The domain ontology used was developed in the context of this study.

Figure 5 is an example of transformation pattern applied in this research study, whose implementation in OPPL2 is shown in Table 4. This pattern defines a histopathology report according to the domain ontology, which contains a set of findings (*hasFinding*), records the total number of adenomas found (*number*) and the size of its biggest adenoma (*maxsize*).

**Table 4:** Definition of the pattern for histopathology reports

?histopathologyReport:INDIVIDUAL,
?finding:INDIVIDUAL,
?size:CONSTANT,
?number:CONSTANT
BEGIN
ADD ?histopathologyReport instanceOf HistopathologyReport,
ADD ?histopathologyReport hasFinding ?finding,
ADD ?histopathologyReport number ?number,
ADD ?histopathologyReport maxsize ?size
END;

#### Chemical compounds

A third use case is currently in progress, although the generation of the semantic dataset has been completed. The objective of this effort is to use semantics to improve compound selection for virtual screening. Virtual screening methods use libraries of small molecules to find the most promising structures that could bind with drug targets. One of such libraries is ZINC [64], a free database of commercially-available compounds for virtual screening. ZINC data can be downloaded in XML format. In this effort, we created the XML Schema and defined the mappings with an ontology developed by our group.

## Discussion

The availability of biomedical datasets in open, semantic formats would facilitate the interoperability of biomedical data and would enable to carry out scientific studies with larger, connected datasets. In this paper we have presented a solution based on the transformation and integration of heterogeneous data resources in which ontologies play a central role. A series of important aspects of our work are discussed next.

### The transformation and integration approach

Our transformation process follows a data warehouse approach instead of an OBDA one because of the following reasons. First, we believe that the availability of RDF/OWL resources is the most natural way of developing the LOD. In our opinion, efforts like Bio2RDF or the EBI RDF platform are correct ways of proceeding for the practical exploitation of biomedical data and the development of the Semantic Web. Second, we are interested in generating OWL knowledge bases over which OWL2 DL reasoning can be applied, which is not ensured by current OBDA approaches. That would be a limitation from our exploitation point of view. We aim to obtain datasets linked to external sources, which is also easier to achieve with our approach. Third, to the best of our knowledge, current OBDA approaches do not facilitate the application of ontology patterns as we do in this work, which also permits a semantically-richer representation and exploitation of data.

Most state-of-the-art transformation approaches and tools from XML or relational databases into RDF/OWL are based on canonical transformations or are not based on mappings with domain knowledge (i.e., ontologies). Such tools mainly perform a syntactic transformation of the traditional formats, making the semantic interoperability of the obtained datasets difficult. Besides, there are no methods that can be applied to both XML and relational databases. Our method provides a semantic representation of the input datasets by performing a transformation guided by domain knowledge using an OWL ontology, so performing an ontology-driven ETL process. This is similar to RDB2OWL [43] and Karma [44]. SWIT and RDB2OWL have in common that the mappings between the input model and the ontology are manually defined, but RDB2OWL is limited to input datasets in relational format and does not provide any solution for the problem of complexity on the manual definition of mappings when using complex ontologies or data integration. Karma has the advantage of performing semi-automatic mapping of input databases and ontologies. However, this mapping process depends on the existence of a knowledge base of previous mappings.

We follow a data warehouse-oriented integration method, although our approach has features associated with the integration based on links, because our mapping rules permit to define links to external datasets. This architecture is similar to the one applied in Bio2RDF, with the difference that our repositories may contain data from multiple sources. Although that could also be possible in the Bio2RDF effort, it is focused on transforming single datasets. In fact, we believe that an effort such as Bio2RDF could benefit from our approach. Currently, one transformation script has to be written to include a new dataset in Bio2RDF. SWIT would reduce the implementation effort for relational or XML sources in the sense that only the definition of the mappings would be needed, since SWIT would execute the data transformation. Besides, SWIT mappings could be reused for new datasets. Using SWIT would have the cost of making explicit the mappings with an OWL ontology, but it would also provide benefits in terms of consistency checking and homogeneity in both the richness of the semantic description and the structure of the data.

Next, some additional aspects concerning the different subprocesses are discussed.

#### Mapping

Data transformation and integration are based on the definition of mappings between the data schema and the OWL ontology. The difficulty and the complexity of mapping not only relies on finding the corresponding entities in the domain ontologies but also on being able to design the corresponding ontology content patterns. Once the rules and patterns are designed, SWIT reduces the implementation effort by executing them and generating the corresponding semantic content. Patterns are also used in Populous [65], which is focused on creating ontologies from spreadsheets. Our experience reveals that semi-automatic mapping techniques would contribute to significantly reduce the mapping time, so efforts in this area are key to support the mapping process.

To the best of our knowledge, there is no standard language to define mappings from different types of input models to OWL ontologies. We are currently using a language based on the former ontology alignment format^22^, which has evolved into EDOAL^23^. The W3C has developed the R2RML mapping language^24^ for mapping relational databases to RDF, but does not cover XML. Our current language permits to express mappings from relational databases and XML schemas to OWL ontologies, and it could be easily extended to cover new types of input models (i.e., OWL ontologies). Our mappings can be reused, especially for data transformation processes that use the same OWL ontology, but the lack of standardisation in this area forces third parties to do some additional work in order to include the mappings generated with SWIT.

In this paper, we have used the mapping rules for creating OWL individuals from XML or relational data, but the process can also be applied for the creation of ontology classes. This might be helpful in case the content of the OWL ontology used is not sufficient for mapping the input schema. For this purpose, the mapping rules were extended to produce OWL classes instead of individuals. This class-based approach also permits to use patterns. The only difference is that the set of variables associated with the pattern are bound to entities instead of instances. We have actually applied such approach for the generation of openEHR archetypes from CEM clinical models [22]. In that study, the input schemas were OWL ontologies corresponding to CEM clinical models and the openEHR OWL ontology was the output schema. Basically, the creation of the openEHR clinical models was approached as extending the openEHR OWL ontology with the specific content of the clinical model. In OWL, being an individual or a class can be seen as the role played by a given concept [66]. The representation of knowledge may therefore be based on individuals or classes, this decision depending on the expected use of such knowledge. In fact, punning was included in OWL 2 DL to enable different uses of the same term, so an individual and a class can have the same URI. From a formal ontology perspective, enabling this possibility might be reason enough for a criticism to our approach, but it is needed from a practical perspective. This situation can be exemplified in the orthology use case. Orthology databases basically contain information about genes, proteins and organisms. In the database they are represented as individuals, but they semantically correspond to classes, since there are many instances of each gene, protein and organism.

#### Transformation

The transformation method checks all the formal aspects that guarantee the generation of consistent content, independently of the use case and the intended exploitation of the data. The logical consistency of the content is guaranteed by the application of OWL reasoning. Consistency is granted independently of the output format, that is, RDF or OWL, because OWL DL semantics is applied during the transformation process. The individuals are expressed in RDF or OWL at the end of the process. In case the methods find that inconsistent content is going to be generated, such content is automatically discarded. Using OWL for ensuring the consistency of the data generated in ETL processes has also been done in other works, such as [42].

Our experience in semantic data transformation in the last years reveals that the semantic representation of the data sometimes needs additional content that is not made explicit in the XML schema or in the corresponding table, so the additional meaning is not provided by the canonical transformation methods. We believe that such additional meaning can be included during the ETL process. In this context, our patterns are equivalent to ontology content patterns [67], which are focused on the definition of templates for the semantically-rich, precise description of the meaning of the content. We believe that ontology content patterns are a solution for those situations in which the source data does not contain all the information needed to generate semantically-rich RDF/OWL content.

The computational complexity of the full method depends on the number of individuals and the mean number of properties and relations per individual. As a consequence, for medium and large datasets, the transformation time may be longer than expected because of the number of instances of axioms to be generated. This might not be a problem in case of stable datasets or batched transformation processes. However, according to our experience with SWIT datasets, the intended exploitation of the dataset might permit to relax some conditions of the transformation process. In case of not performing integration processes, identity rules are only needed if, for instance, two entities from the input are mapped onto the same ontology class. In case of not requiring automated reasoning on the transformed dataset, the generation of some types of axioms might be omitted, saving time and space. *owl:differentFrom* axioms are an example of a time and space consuming type of axiom, but they might be skipped in some cases. The lesson learned here is that the optimal configuration of transformation depends on the use case, so the flexibility of the process is basic for getting the desired semantic dataset. All these aspects can be considered adjustable parameters for the execution of the process using tools like SWIT.

#### Integration

The integration method is useful for scenarios that require the creation of a repository that includes portions of data from different resources. In case of wishing a link-based integration, the mechanism offered by SWIT to include links in the mapping rules could be sufficient. The key objectives of the integration method are (1) detecting equivalent data instances to reduce redundancy and (2) ensuring the consistency of the resulting repository. Both tasks are supported by OWL reasoning.

Identity rules are fundamental in the integration process, because they control the redundancy of the individuals created. They describe which properties permit identifying an individual of a certain ontology class. For example, we could integrate two resources about proteins which use different identifiers for the proteins, and those identifiers are used in the URI of the individual. Those resources might be using the Human Genome nomenclature for naming the gene associated with the protein. If the gene name is used in the identity rule, then SWIT would find that both individuals refer to the same protein.

In addition to this, the meaning of our identity rules is similar to the identity criteria proposed by formal ontologists [68], because they determine which conditions are sufficient for identity. The properties used in identity rules are those that would be included in OWL Key axioms^25^. Key axioms associate a set of object properties and datatype properties with a class, so each individual is identified by the values of such set of properties. Hence, when two individuals have the same values for such properties, they are considered the same individual. Key axioms are only applied over those individuals with asserted membership to a class. Such inferencing-related limitation made us to define our identity rules.

#### Interoperability

Next, we discuss how and to what extent SWIT promotes or facilitates the interoperability of the datasets. Let us consider a resource about proteins, which uses a local URI for each protein, but also stores the UniProt Accession Numbers (AC). Let us suppose that we want to link every protein to the corresponding URI in UniProt. It should be noted that the UniProt URI for each protein differs in the AC. For example, the URI for the protein *P63284* is http://purl.uniprot.org/uniprot/P63284. SWIT provides two different ways for creating such link:

1. Redefinition of the URI. We can use the UniProt URI instead of the dataset URI, since SWIT permits to define which prefix has to be used in the transformation of each entity.
2. Linkage of resources with *owl:sameAs*. The transformation of the protein uses the dataset URI but creates an *owl:sameAs* link to the UniProt URI.

Either action can be applied to transform the data with cross-references to external resources, but this requires to know which external resources will be used during the definition of the mappings. Additional research on the identification of related datasets would permit our method to evolve to generate richer Linked Open Datasets.

Let us revisit now the example introduced in the integration subsection, whose goal is to integrate data about the protein A from two resources which have different identifiers for that protein. Let us suppose that the transformation and integration are driven by an OWL ontology which contains a class Protein. Generally speaking, these are the possible situations:

1. The class Protein has an identity rule associated, and the properties of the identity rule have the same value for protein A in both resources.
2. The class Protein has an identity rule associated, but the properties of the identity rule have different value for protein A in both resources.
3. The class Protein does not have an identity rule associated.

In the case 1, the definition of the transformation scenario in SWIT will determine whether (i) the two individuals are linked through *owl:sameAs*; or (ii) they are merged into a single individual. In the case (i), the two instances of Protein A would co-exist, but *owl:sameAs* would facilitate interoperability. This would be an appropriate decision if having RDF versions of each source dataset is desired. The integration capabilities offered by SWIT would facilitate the creation of those links if all the data are stored in the same repository. The decision of whether merging or linking is done in the behaviour of the identity rule. In the case (ii), a new decision has to be made, since each instance of Protein A would have a different URI, but the merged instance would have one URI. For those cases, SWIT permits to use either the URI of one of the instances or a different prefix to which an identifier would be added. This is also included in the behaviour of the identity rule.

In the cases 2 and 3, SWIT would generate two different individuals, since the sufficient conditions for identity are not met. The current version of SWIT does not discover equivalent instances, which is considered further work. Discovery methods should be carefully used, since the identity rules defined in the ontology should be respected.

Consequently, we believe that SWIT contributes to the interoperability of datasets, since it includes mechanisms for unifying URIs and defining links between URIs that represent the same entity, especially when all these resources are transformed and integrated in a common repository. Further research will permit to manage SWIT integration processes in distributed repositories.

### The SWIT datasets

The mapping rules, including the patterns, permit to transform input data into RDF/OWL, which are the formats used by the Semantic Web community for the development of the Web of Data and the Linked Open Data cloud. SWIT permits to achieve five-stars data repositories, because our method permits to include links to external resources in the mapping rules for integration and interoperability purposes.

The datasets generated using our method have demonstrated their usefulness in the related studies. In the case of orthology data, the heterogeneity of the orthology-related datasets suggested us to extend the work done in OGOLOD. OrthoXML is the most popular format for representing orthology data. We have recently reused our work to define a canonical mapping from OrthoXML to the domain Orthology Ontology (ORTH), so we are providing a means for generating open datasets to the orthology community. Each OrthoXML resource could be automatically transformed and exploited jointly with the rest of content. In the case of EHR data, the rules for classifying the patients by level of risk of developing colorectal cancer were implemented in OWL. This effort permitted to develop a semantic infrastructure for classifying patients by levels of risk, which could be reused for other source datasets by just adapting the mapping rules. This would also permit the joint exploitation of different datasets transformed to our semantic infrastructure. Therefore, the use of such approach permits to create reusable and extensible datasets, which is a feature needed for biomedical research datasets. Another lesson learned is that the same approach can be applied for generating public or private datasets, since they only differ in their access policy.

## Conclusions

In this paper we have presented an approach that is able to generate open biomedical repositories in Semantic Web formats. The generation process is based on the ontology-driven transformation of data and integration is performed following Semantic Web principles. The method has been implemented in the SWIT tool, which automates and standardises the process of generating five stars, semantic, open biomedical datasets. We believe that the approach (1) enables the common transformation and integration process for heterogeneous biomedical data; (2) permits the design of reusable mappings driven by domain knowledge; and (3) applies Linked Open Data principles to generate interoperable, semantically-rich, open, biomedical datasets. Future work will address the assistance on the recommendation of semi-automatic mappings and on providing more flexibility in the transformation process.

## Endnotes

^1^http://linkeddata.org/

^2^http://www.w3.org/RDF/

^3^http://www.w3.org/TR/owl-features/

^4^http://www.openehr.org/releases/1.0.2/architecture/am/adl.pdf

^5^http://www.w3.org/TR/rdf-sparql-query/

^6^http://www.w3.org/DesignIssues/LinkedData.html

^7^http://rhizomik.net/html/redefer/xml2rdf/

^8^http://rhizomik.net/html/redefer/xsd2owl/

^9^http://www.w3.org/2001/sw/rdb2rdf/

^10^https://www.w3.org/Submission/xsparql-language-specification

^11^https://www.w3.org/Submission/SWRL

^12^http://purl.org/net/ORTH

^13^https://github.com/oborel/obo-relations

^14^http://purl.bioontology.org/ontology/NCBITAXON

^15^http://purl.bioontology.org/ontology/CDAO

^16^https://code.google.com/p/semanticscience/wiki/SIO

^17^http://oppl2.sourceforge.net/

^18^http://sele.inf.um.es/swit

^19^http://jena.apache.org

^20^https://github.com/qfo/OrthologyOntology

^21^http://questfororthologs.org/

^22^http://alignapi.gforge.inria.fr/format.html

^23^http://alignapi.gforge.inria.fr/edoal.html

^24^http://www.w3.org/TR/r2rml/

^25^http://www.w3.org/TR/owl2-syntax/#Keys
